# Inequalities in maternal malnutrition in Ethiopia: evidence from a nationally representative data

**DOI:** 10.1186/s12905-020-01154-8

**Published:** 2021-01-02

**Authors:** Nigatu Regassa Geda

**Affiliations:** Center for Population Studies, College of Development Studies, Addis Ababa University, Sidist Kilo Campus, PO Box 1176, Addis Ababa, Ethiopia

**Keywords:** Inequalities, Obesity, Undernutrition, Body mass index

## Abstract

**Background:**

Despite promising progress made in several maternal health indicators, maternal malnutrition (especially undernutrition) remained one of the greatest development challenges for Ethiopia. The main purpose of this study was to examine the disparities in maternal malnutrition and estimate the population level impacts of key risk factors in Ethiopia.

**Methods:**

The analysis was made based on the Ethiopian Demographic and Health Survey (EDHS 2016) data, containing 9949 non-pregnant women. Multiple logistic regression was used to examine the effects of selected explanatory variables on the two nutrition morbidity outcomes (undernutrition and obesity). Two population weighed regression-based measures (the Slope Index of Inequality/SII and Relative Index of Inequality/RII) and Population Attributable Fractions (PAFs) were employed to examine the inequalities in maternal malnutrition.

**Results:**

The prevalence of maternal undernutrition and overweight or obesity were 21% and 6%, respectively. In the multiple logistic regression, four variables appeared to have significant association with both undernutrition and overweight/ obesity, namely age of the women, residence, maternal education, and non-monetary wealth (*p* < 0.05). Both the absolute and relative measures of inequalities showed remarkable differences in maternal undernutrition, significantly favoring the higher socioeconomic groups (*p* < 0.05). Further analysis of inequalities, using the Population Attributable Fractions (PAFs), revealed that the combined population level impacts of all the significant risk factors amount 80.38%, from which 25% is attributed to the three socioeconomic variables (non-monetary wealth, maternal education and paternal education).

**Conclusion and policy implication:**

Given the high disparity in both under nutrition and overweight and/or obesity, the study emphasized the need for policy and program efforts to promote parental education in Ethiopia. Strengthening nutrition sensitive mass literacy programs is recommended along with improving women’s employment and rural households’ income for increased access to better diet.

## Background

Maternal malnutrition, which refers to undernutrition and overweight/obesity, is one of the most important public health concerns in Asian and sub-Saharan African countries. In some Sub Saharan African countries maternal undernutrition is affecting up to 40% of women [1, 2]. Malnutrition among mothers is commonly measured by Body Mass Index (BMI, weight (kg)/height (m^2^)) [3]. Mothers with BMI < 18.5 is defined as undernutrition [3].

The health impacts of maternal malnutrition are reported in several studies. For instance, malnutrition is known to have intergenerational effects [4] and increases the risk of poor pregnancy outcomes such as premature or low-birth-weight (LBW) babies, obstructed labor and postpartum hemorrhage [4–6]. Overweight and obesity are also positively associated with risk of diabetes, hypertension and other health complications [6].

Inequalities in maternal malnutrition occur due to several social, demographic, and economic factors. Education and income-based inequalities are commonly reported parameters [7–9]. Studies in some African countries [8, 10] reported inequalities in maternal malnutrition by showing that mothers having some education had much lower prevalence compared to those with no education [8, 10]. Inequalities are also attributed to differences in other individual characteristics (such as level of decision making autonomy about reproductive health and nutritional matters, work status, etc.) and household level characteristics [10].

Common forms of malnutrition in Ethiopia include acute and chronic undernutrition, vitamin A deficiency (VAD), iron deficiency anemia (IDA), and iodine deficiency disorder (IDD) [11]. Low dietary intake, inequitable distribution of food within the household, improper food storage and preparation, dietary taboos, and infectious diseases characterize most households in Ethiopia [12]. Despite improvements in several health and development indicators in recent years [13], Ethiopia remained as one of the countries with the highest prevalence of maternal undernutrition. The rate of undernutrition declined from 30.5% in 2000 to 22% in 2016 [12].

With an effort to alleviate the widespread malnutrition in the country, the government of Ethiopia devised a National Nutrition Strategy (NNS) [14], and launched a National Nutrition Program (NNP) since 2008 [14]. It incorporated promotion of Essential Nutrition Actions (ENA) as a strategy in view of improving the nutritional status of women and children [15]. The main challenges in this endeavor is that more than half of Ethiopian women are non-educated, most of whom are living in poorer and poorest households, and nearly 85% living in rural areas with little access to services [12]. This has resulted in huge inequalities in selected maternal health outcomes [16].Thus, the situation necessitates evidence-based findings about the growing socioeconomic disparities to support the ongoing efforts and intervention programs. To date, to the best of author’s knowledge, there are no comprehensive studies conducted on inequalities and population level impacts of key risk factors of maternal malnutrition in Ethiopia. Therefore, the main objective of this study is to examine the disparities in maternal malnutrition in Ethiopia based on the most recent nationally representative data.

## Methods

### The study context

Ethiopia has a Federal system with nine autonomous Regional States. With an estimated population of 112 million people, the country is dominantly a young population with rapid annual growth rate (about 2.5% per annum). This makes the country the second most populous country in Africa [17]. Ethiopia is the least urbanized country in the world, and has an agrarian economy, where agriculture accounts for more than 60% of the GDP and employs nearly 85% of the population [18]. Severely affected by poverty, food insecurity and communicable diseases, rural women are highly exposed to undernutrition and micronutrient deficiencies [19]. The country experiences one of the highest incidences of child and maternal nutritional deficiencies which contribute to increased morbidity and mortality [12].

### Data sources

This study is based on a nationally representative cross-sectional data from the Ethiopian Demographic and Health Survey/EDHS, conducted in 2016 (CSA and ICF, 2016). The EDHS collected sociodemographic data from 10,641 mothers with under five children. The present study used weighted samples of 9949 mothers who were not pregnant at the time and who were aged 15–49 years. The data file contained a wide range of woman’s socioeconomic and demographic variables including key characteristics of her husband.

### Ethics statement

EDHS followed previously approved standard protocols, data collection tools and procedures. Participation in the survey was voluntary [12]. Permission to use the data for the purposes of the present study was granted by ICF (U.S.) and Central Statistics Authority (Ethiopia). The ICF and CSA took informed consent from respondents prior to the administration of the questionnaire. Also, written informed consent was obtained from a parent or guardian for participants under 16 years old.

### Measures of outcome and exposure variables

The main outcome variables are maternal undernutrition and overweight and/or obesity. Maternal malnutrition was computed as BMI, the ratio of weight (kg) and the square of height (m). Mothers with BMI < 18.5 were defined as having undernutrition. Those with value of 25–29.9 and > 30 were defined as overweight and obese, respectively [3, 20]. Information on parental education was measured as the reported number of years of maternal/paternal education and then allocated within conventional educational categories (e.g., no education, primary, secondary, and post-secondary education). Given the difficulty in generating data on actual household income, EDHS constructed a wealth index from selected key household assets including household ownership of consumer goods, dwelling materials, sources of drinking water, types of sanitation facilities, and other characteristics that relate to economic status [12]. The wealth index was computed by using the Principal Component Analysis (PCA) [21]. This method assigns a weight or factor score, and then standardizes and sums of the scores for each household. The entire sample was then ranked and divided into successive quintiles from the first quintile (Q1 = the poorest 20% of the household population) to the fifth quintile (Q5 = the richest 20% [12, 21].

Other than the main exposure variables described above, seven individual, /household/ community level characteristics were included in the analysis as confounding variables. These were age (15–24, 25–34 and 34 + years), type of residence (rural vs urban), religion (Orthodox Christian vs others), type of family structure (monogamous vs polygamous), work status (working vs not working), household size (small, large and medium), and household headship (male vs female).

### Statistical analysis

Descriptive analysis was used to examine the characteristics of the sample. Bivariate logistic regression was conducted to select the variables with *p* values < 0.2. Multiple logistic regression analyses were conducted to examine the association between selected predictors and the two maternal nutritional morbidity variables, adjusting for confounders. The logistic regression model is given by the equation.

1 $${\text{p}}/\left( {{1} - {\text{p}}} \right) = {\text{exp}}\left( {{\text{a}} + {\text{Bx}} + {\text{c}}} \right)$$

where P is the probability that the event y occurs, at p(y = 1); and p/[1−p] is the “odds ratio”. We used a *p* ≤ 0.05 to ascertain statistical significance [22]. Hosmer–Lemeshow test was used to check the goodness of fit in our final model[23]. We used STATA 13 for data management, and data were weighted for descriptive analysis using DHS recommendation.

Inequalities in maternal malnutrition was estimated using a combination of regression-based absolute and relative measuring tools, namely Slope Index of Inequality (SII), Relative Index of Inequality (RII) and Population Attributable Fractions (PAFs). The SII is an absolute measure of the difference of inequality among socioeconomic groups within of a population of interest. The RII is a relative measure, derived from the SII, and considers the size of the population and the relative disadvantage experienced by different subgroups [24]. The computation of the SII and RII started with computing the prevalence of maternal undernutrition and overweight/obese by socioeconomic subgroups (wealth and parental education). Scores were then assigned based on the midpoint range in the cumulative distribution within the population. The SII were estimated by Weighted Least Square (WLS) regression considering the relative rank in the cumulative distribution of the wealth and parental education [24]. The SII is the linear regression coefficient or slope of the regression line given by:

2 $${\text{Y}}_{{{\text{ij}}}} = \beta_{{0{\text{j}}}} + \beta_{{{\text{1j}}}} {\text{X}}_{{{\text{ij}}}} + {\text{e}}_{{{\text{ij}}}}$$

where Y_ij_: the mean value of overweight/obesity, X_ij_: the relative rank of the wealth quantile I, β_0j_: the slope showing the relationship between a group’s and its relative socioeconomic rank. e_ij_: is the error distribution/unexplained error.

The fact that we used socioeconomic groups (analogous to individual ranking data), the regression error term in these Ordinary Least Square (OLS) model presented above becomes less reliable in terms of fulfilling the heteroscedasticity assumption. The relative index of inequality, RII, is derived from SII and the population mean (µ) of the health outcome, given by:

3 $${\text{RII}} = {\text{SII}}/\mu = \beta /\mu$$

Population Attributable Fractions (PAFs) were used to estimate the inequalities in undernutrition across several risk factors to assess the burden at the population level. We used logistic regression estimates to get adjusted PAFs [24–26].

The PAFs are directly obtained from logistic regression which was introduced by Greenland and Drescher[25]. The basic idea behind this approach is to estimate a logistic regression model with all known/available risk factors. Ruckinger et al. [26] provided the following steps for calculating the PAF of the risk factor of interest: a)The risk factor has to be coded dichotomously, and then 'removed' from the population by classifying all individuals as unexposed, irrespective of their real status b) predicted probabilities for each individual should be calculated using this modified dataset, given by pp = 1/ 1 + exp(−α + β x_i_); where α represents the estimate for the intercept of the logistic regression model, β denotes the parameter vector for the covariates included in the model, and xi denoting the observations of the covariates for each individual, however, with the 'removed' covariate set to zero for all individuals c) Computing the adjusted number of cases of the disease (i.e. overweight or obesity) that is obtained by summing up all predicted probabilities that would be expected if the risk factor was absent in the population, and d) the PAF is then calculated by subtracting these expected cases from the observed cases and dividing by the observed cases[26].

## Results

### Participant characteristics

Table 1 summarizes the characteristics of the 9638 non-pregnant women in the reproductive age group (15–49 years). Over half (52.7%) were in the main childbearing ages of 24–34 years. The mean age of mothers was 29.37 years (SD of 6.62). Close to 12% of the women were living in rural areas. One quarter (27%) of women were generating income from gainful work, and more than two-thirds were housewives. Two-thirds of the mothers and 51.2% of fathers had no education, with only 7.4% and 11.7% of mothers and fathers (respectively) were in the secondary and above levels. More than half of the respondents lived in medium size households [4–6members], and many (39.7%) were living in large size households (7 + members). More of the mothers were living in poorest/poorer households (45%) compared to those living in richest/richer households (33.3%). Close to 11% of the women were living in a polygamous marriage.

**Table 1:** Background characteristics of female respondents of child-bearing age, N = 9949

Characteristics	N	%
*Age of the mother*		
15–24	2168	21.8
25–34	5245	52.7
34 +	2536	25.5
*Place of residence*		
Urban	1147	11.5
Rural	8802	88.5
*Work status*		
Working	2725	27.4
Not working	7224	72.6
*Education of mothers*		
No education	6581	66.1
Primary level	2636	26.5
Secondary and above	732	7.4
*Education of fathers*		
No education	5092	51.2
Primary	3691	37.1
Secondary and higher	1166	11.7
*Household size*		
1–3 members	961	9.7
4–7 members	5037	50.6
7 +	3951	39.7
*Wealth index*		
Poorest	2335	23.5
Poorer	2236	22.5
Middle	2074	20.8
Richer	1796	18.1
Richest	1508	15.2
*Family structure*		
Monogamous	8314	83.6
Polygamous	1082	10.9

### Determinants of malnutrition

Table 2 presents the bivariate logistic regression for nine potential predictors selected based on review of literature. Since all variables had *p* values < 0.2, they were all considered for further analysis in the multiple logistic regression model.

**Table 2:** Bivariate logistic regression for selected predictors vs maternal undernutrition and overweigh/obesity, N = 9949

	Model 1: Undernutrition			Model 2: Overweight/obesity		
	Adjusted OR	95%CI	*P* value	Adjusted OR	95%CI	*P* value
*Age of the women*						
15-24^RC^						
25–34	0.687	0.595–0.793	< 0.001	2.357	1.827–3.043	< 0.001
34 +	0.739	0.622–0.878	0.001	2.367	1.804–3.107	< 0.001
*Place of residence*						
Urban^RC^						
Rural	2.456	1.977–3.051	< 0.001	0.129	0.104–0.158	< 0.001
*Household size*						
0–3^RC^						
4–6	0.891	0.753–1.054	0.180	0.820	0.645–1.044	0.108
7 and above	0.872	0.728–1.046	0.140	0.879	0.689–1.118	0.290
*Education of mothers*						
No education^RC^						
Primary level	0.798	0.693–0.919	0.002	1.647	1.326–2.045	< 0.001
Secondary and above	0.633	0.504–0.793	< 0.001	5.149	4.133–6.414	< 0.001
*Education of father*						
No education^RC^						
Primary level	0.777	0.668–0.903	0.001	1.135	0.903–1.428	0.277
Secondary and above	0.685	0.577–0.814	< 0.001	3.614	2.909–4.490	< 0.001
*Religion*						
Orthodox Christians^RC^						
Muslims	1.336	1.145–1.558	< 0.001	0.901	0.728–0.114	0.336
Others	1.221	0.997–1.495	0.053	0.498	0.376–0.659	< 0.001
*Wealth status*						
Poorest^RC^						
Poorer	0.615	0.511–0.739	< 0.001	1.064	0.720–1.572	0.754
Middle	0.619	0.512–0.749	< 0.001	0.855	0.559–1.305	0.468
Richer	0.508	0.418–0.618	< 0.001	1.359	0.915–2.019	0.128
Richest	0.271	0.219–0.334	< 0.001	8.594	6.524–11.320	< 0.001
*Women’s work status*						
Not working^RC^						
Working	1.253	1.092–1.437	0.001	0.664	0.559–0.789	< 0.001
*Household headship*						
Male^RC^						
Female	1.081	0.920–1.269	0.143	1.423831	1.157–1.751	0.001

RC, reference category

Multivariable logistic regression analyses were carried out to determine factors associated with malnutrition among women of reproductive age in the study population. Table 3 presents the findings for both undernutrition and overweight/obesity under two separate models. Four variables appeared to have significant association with both undernutrition and overweight/ obesity, namely age of the women, residence, maternal education, and non-monetary wealth. It is seen that odds of undernutrition decreases for mothers aged 25–34 years by 27.4% (AOR = 0.726, 95% CI 0.638–0.827) compared to younger women aged 15–24 years. It appeared that mothers living in rural households were 1.65 times more likely to be undernourished compared to those living in urban households (AOR = 1.651, *p* < 0.001). However, the odds decrease for overweight/ obesity by 63.2% (AOR = 0.368, *p* < 0.001) for women living in rural areas compared to those residing in urban areas. The odds of maternal undernutrition decreases with household size where those living in household size of 4–6 and 7 + were 28.2% and 26.9% less likely to be undernourished. The odds of undernutrition and overweight/obesity decreases by 17.5% and 55.5% (AOR = 0.825 and AOR = 0.445) for women with primary level of education compared to those with no education. Fathers education appeared to be significant predictor only in model 2 (overweight/obesity) where the prevalence decreases by 42.3% for fathers with primary education compared to those with no education (AOR = 0.577, *p* < 0.05). Women of Muslim affiliations were 1.336 times more likely to become overweight/obese compared to Orthodox Christians. The odds of undernutrition decreases for women living in richer and richest households by 15.7% and 30.8%, respectively (AOR = 0.843 and AOR = 0.692) compared to those living in poorest households. On the contrary, women living in richer and richest households are 1.53 and 3.97 times more likely to become overweight/obese compared to those living in poorest households.

**Table 3:** Multiple logistic regression for selected predictors vs maternal undernutrition and overweigh/obesity, N = 9949

	Model 1: undernutrition			Model 2: overweight/obesity		
	Adjusted OR	95%CI	P Value	Adjusted OR	95%CI	P Value
*Age of the women*			< 0.001			< 0.001
15-24^RC^						
25–34	0.726	0.638–0.827	< 0.001	2.824	2.036–3.918	< 0.001
34 +	0.880	0.752–1.031	0.113	3.002	2.076–4.342	< 0.001
*Place of residence*						
Urban^RC^						
Rural	1.651	1.286–2.121	< 0.001	0.368	0.271–0.0.500	< 0.001
*Household size*			0.003			< 0.001
0–3^RC^						
4–6	0.772	0.653–0.913	0.002	0.775	0.567–1.059	0.110
7 and above	0.731	0.607–0.880	0.001	1.242	0.878–1.755	0.220
*Education of mothers*			0.004			0.015
No education^RC^						
Primary level	0.825	0.727–0.935	0.003	1.442	1.125–1.849	0.004
Secondary and above	1.052	0.810–1.365	0.704	1.313	0.929–1.854	0.122
*Education of father*			0.273			< 0.001
No education						
Primary level	0.943	0.845–1.052	0.292	0.577	0.450–0.741	< 0.001
Secondary and above	0.856	0.697–1.052	0.139	1.218	0.908–1.635	0.189
*Religion*			< 0.001			0.041
Orthodox Christians^RC^						
Muslims	1.101	0.985–1.230	0.090	1.336	1.067–1.673	0.012
Others	0.647	0.565–0.741	< 0.001	1.157	0.905–1.480	0.244
*Wealth status*			0.001			< 0.001
Poorest^RC^						
Poorer	0.784	0.684–0.898	< 0.001	0.886	0.611–1.286	0.525
Middle	0.0.821	0.714–0.944	0.006	0.832	0.566–1.224	0.351
Richer	0.843	0.725–0.979	0.025	1.527	1.078–2.162	0.017
Richest	0.698	0.555–0.876	0.0.002	3.973	2.742–5.758	0.000
*Women’s work status*						
Not working^RC^						
Working	0.932	0.834–1.042	0.0.218	0.0.823	0.673–1.006	0.057
*Household headship*						
Male^RC^						
Female	1.118	0.968–1.291	0.129	0.813	0.642–1.030	0.086
Constant	0.385		< 0.001	0.037		< 0.001

RC = reference category

### Inequalities in maternal malnutrition

Table 4 presents the results of the regression based SII and RII for inequalities in both maternal undernutrition and overweight/obesity. The findings show that parental education has significant (*p* < 0.05) effect on both outcome variables. As the SII values are the beta (b) values of each individual weighted linear regression equation, and they represent the effect of a variable on the outcome as we move from the lowest to the highest socioeconomic group (i.e. within the wealth quintiles and education groups). The negative SII and RII values, therefore, indicate a decrease in maternal undernutrition and overweight/obesity as we move from the lowest to highest education or wealth groups.

**Table 4:** Absolute and relative measures of inequity for maternal malnutrition in Ethiopian N = 9949

	Absolute effects			Relative effects		
Disparity variable	SII	CI		RII	CI	
		Lower	Upper		Lower	Upper
*Maternal undernutrition*						
Non-monetary wealth	− 19.593**	− 26.953	− 12.232	− 0.979**	− 1.348	0.611
Maternal education	106.062***	− 114.938	− 97.187	− 5.303***	5.747	− 0.4.60
Paternal education	− 71.452**	− 143.238	− 2.334	− 3.573**	− 7.162-	− 0.617
*Overweight/ obesity*						
Wealth based	22.149	− 39.700	83.997	1.107	− 1.985	4.200
Maternal education	− 46.657**	− 83.154	− 10.161	− 2.333**	− 4.158	0.508
Paternal education	− 20.756**	− 29.219	− 12.293	− 1.038**	− 1.461	0.615

*p < 0.05, **p < 0.01, **p < 0.001

It is seen in Table 4 that the prevalence of maternal undernutrition significantly decreased by nearly 20% as we move from wealth quintile 1 (poorest) to 5 (richest). In relative measures, it implies that the prevalence of undernutrition decreased by 98% (RII = −0.979) as one moves from wealth quintile 1 (poorest) to 5 (richest). The absolute effects of mothers’ education on the prevalence of maternal malnutrition was significant (*p* < 0.001). Notably, there was a decrease in the prevalence of maternal undernutrition by 106 percentage points as we move from mothers with no education to higher education. In relative measure, this implies a 530% decrease in the prevalence (RII = −5.303) as we move from mothers with no education to those with higher education. Similarly, the prevalence of maternal overweight/obesity decreased by 47% as we move from mothers with no education to those with higher education. In relative measure, the decrease in the prevalence amounted 223% (RII = −2.233). The effects of paternal education on the prevalence of maternal malnutrition was significant (*p* < 0.01). The prevalence significantly decreased by 71% and 21% for undernutrition and overweight/obesity respectively, as we move from fathers with no education to those with higher education. In terms of relative measure, the prevalence decreased by 357% (RII = −3.573) and 104% (RII = −1.038) for undernutrition and overweight/obesity, respectively.

**Fig. 1:**
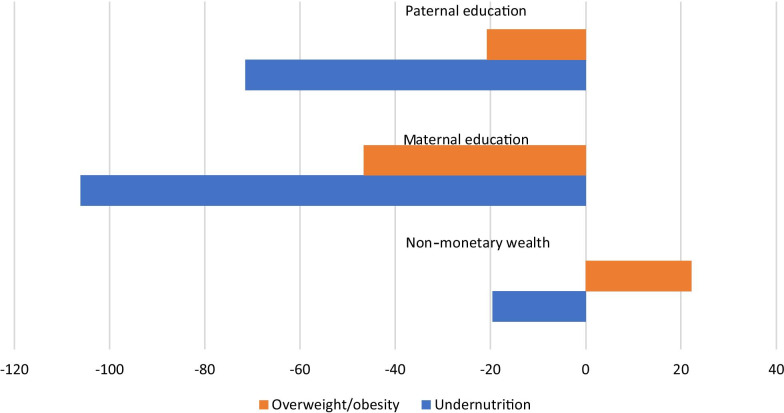
Socioeconomic inequality in malnutrition, Ethiopia*, *Figure is derived from Table 4 data using the SII values

Figure 1 further shows that inequalities in undernutrition is more pervasive than in obesity/overweight. Compared to wealth based inequalities, the magnitude of parental education based inequalities were stronger (see Fig. 1).

Table 5 presents the population estimates of attributable fraction as an impact measurement of an individual risk factor at the population level. Due to small proportion of obese/overweight women, the PAFs were computed only for undernutrition. The estimated adjusted PAFs indicate that place of residence contributed the biggest proportion (39.64%) of impacts. This is interpreted as the proportion of undernourished women that could have been prevented at the population level if exposure (living in rural) is removed. Likewise, 13.32% of the undernourished mothers could have been prevented in the absence of non-educated mothers. Age of women, religion, wealth status and family structure were also important risk factors whose combined impact at population level is close to 28%. The combined population level impacts of all the significant risk factors amount 80.38%.

**Table 5:** Population Attributable Fractions (PAFs) for selected risk factors of maternal undernutrition, N = 9949

Risk factors	OR (95% C.I) adjusted	PAF (adjusted) %
Age of the women (15–24 years)	1.439 (1.278–1.621)	7.93
Place of residence (rural)	1.743 (1.444–2.105)	39.64
Religion (Orthodox Christian)	1.133 (1.024–1.254)	7.51
Education of mothers (no education)	1.231 (1.091–1.388)	13.32
Education father (no education)	1.108 (1.198–1.230)	5.46
Wealth status (poorest)	1.274 (1.140–1.425)	6.23
Women’s work status (not working)	1.026 (0.875–1.204)	1.85
Family structure (monogamous)	1.337 (1.203–1.487)	5.75
Headship (female headed household)	1.067 (0.924–1.233)	1.43
Total for all variables		89.12
Total for only significant variables		80.38

## Discussion

The current study examined the risk factors and inequalities in maternal malnutrition in Ethiopia based on a nationally representative data. We found that the prevalence of undernutrition among mothers was 22%, which showed a decline of 8 percentage points from the year 2000 Ethiopian Demographic and Health Survey (30%)[27]. The prevalence of overweight or obesity was about 8% which increased by 5 percentage points from the year 2000 [27]. The mean BMI for all the women in this sample was 21. The prevalence of undernutrition was high compared to recent national reports from other African countries. For instance, a recent national study in Madagascar reported the prevalence of maternal undernutrition and overweight/obesity as 17% and 5.8%,, respectively [28]. A review of the most recent Demographic Health Surveys (DHS) for East and South African countries suggests lower prevalence of undernutrition (14%) and higher prevalence of overweight/obesity (15%)[28].

The non-monetary wealth significantly predicted both undernutrition and overweight/obesity in the multiple logistic regression. Also, both the absolute and relative measures further confirmed remarkable wealth inequalities in maternal undernutrition significantly favoring the higher socioeconomic groups. This finding is consistent with a recent evidence confirming rapidly increasing maternal overweight and increased inequality in the prevalence of maternal undernutrition in rural areas and indigenous/socially disadvantage communities [29].

The multivariate analysis witnessed significant association between the maternal education and the prevalence of both undernutrition and overweight/obese. The likelihood of being undernourished and overweight/obese was lower for those with primary level of education. The result did not show significant for mothers with secondary and above education, due mainly to smaller number of women falling in this category. Both the absolute and relative measures further confirmed remarkable inequalities in both outcomes favoring the higher socioeconomic groups. There is no doubt that lack of formal schooling poses challenges on knowledge reception capacity and translation of interventions directed to improve maternal and child nutrition [30–31]. This implies that women with even a minimal education are generally more aware of their own nutritional status and that of their families [32] as education may provide them some decision making autonomy and greater access to household resources that are important to nutritional status [9]. Recent studies from South East Asian countries reported that women with no education and those residing in rural areas are at higher odds of being underweight compared to those who are more educated and living in urban areas [33–36]. Earlier comparable studies conducted in Ethiopia reported an inverse relationship between women’s education and level of undernourishment among women [37–38]. Women’s education enhances their income generation ability their autonomy and help them develop greater confidence and capability to make decisions about their own and children’s health [39].

Findings on effects of paternal education on inequalities in both maternal undernutrition and overweigh/obese is rarely reported by other studies. It can be argued that the effects of fathers’ education on inequality in maternal undernutrition may not necessarily be direct. Studies show that educated fathers are more likely to significantly contribute to maternal health outcome through increased family income, provide more freedom and choices to their wives, family social status and stability, opportunities for children to access health care, availability of social support, and other aspects of the household ecology [39–41]. In a recent national level study in Bangladesh, Nguyen et al. [33]. reported that the log odds of maternal dietary diversity increased by 24 units for every unit increase in the support level of husbands.

Further analysis of the risk factors using the PAFs confirmed the combined population level impacts of the three socioeconomic variables, along with other variables, on maternal undernutrition. It is noted that about 80% of occurrence of maternal undernutrition was attributed to eight risk factors including rural residence, non-educated mothers, non-educated fathers, being young (age 15–24 years), religion (Orthodox Christian), being in poorest wealth category, and living in polygamous family structure. The combined population level impacts of the three socioeconomic variables (non-monetary wealth, maternal education, and paternal education) make up 25%. It means that controlling these known conventional risk factors could potentially prevent about a quarter of the reported burden of maternal undernutrition in the population. Though comparison of the reported PAFs with reports from other studies in different setting is difficult mainly due to varying prevalence of risk factors [26], the findings broaden our understanding of the impacts of the risk factors on maternal malnutrition in the population.

From the analysis and discussions presented above, it turns out that the three socioeconomic variables (poor maternal and paternal education and low non-monetary wealth status) along with other modifiable risk factors have significant impacts on the maternal malnutrition. They, therefore, deserve more attention and considerations of the policy makers and regional administrators. Policy and program efforts that promote women’s basic education and exceeding primary education level are important to reduce undernutrition. Mass literacy programs containing nutrition messages must be strengthened for building the capacities and knowledge of rural women. Improving women’s employment opportunities would increase the household’s income, thereby increases households’ nutritional status.

### Strengths and limitations

The current study has explored multiple socioeconomic inequalities in maternal malnutrition in Ethiopia. The results will undoubtedly contribute to the limited available literature in the field and serve as a reference points for future researchers and policy makers. The findings may be useful for national level planning, targeting, monitoring, and evaluating nutrition programs. It also provides some insight into the growing problem of overweight/obesity among adult women. However, this study is not without limitations. First, due to its cross-sectional nature, where information was collected at a specific point in time in the respondents’ life, it is difficult to draw temporal relationship between the exposure and outcome of interest. Second, because about two third of the women respondents had no education, there might be under reporting of some significant variables such as age, pregnancies and birth and other sociodemographic variables used in this study. Third, some of the factors that were not captured in the multivariate regression model might have influenced the estimated level of inequalities in the PAFs as the accuracy of these estimates also depends on the completeness of the multivariable model. Such variables may include a range of political, economic, technological, and cultural factors that exert some impact on the overall level and distribution of maternal malnutrition at community/ country level. Finally, the impacts of some of the risk factors generated from an adjusted AF model (such as age and religion) are less modifiable risk factors in short run.

## Conclusion

Given the unacceptable high maternal undernutrition and slowly creeping rate of overweight/obesity, the current study emphasizes on the importance of addressing both the demand side (such as provision of economic support to poor rural women, education and health promotion activities, improving access to healthy diet) and supply side interventions such as strengthening policies and programmes that can simultaneously reduce the risk or burden of both undernutrition and overweight/obesity in the country.

## Abbreviations

BMI: Body mass index
CSA: Central Statistics Authority
DHS: Demographic and Health Surveys (DHS)
EDHS: Ethiopian Demographic and Health Surveys
SDGs: Sustainable Development Goals
WHO: World Health Organization
